# Supported employment coaches’ difficulties and facilitators with clients diagnosed with personality versus other disorders: A qualitative study

**DOI:** 10.1016/j.heliyon.2024.e32955

**Published:** 2024-06-13

**Authors:** Noëllie Dunand, Marine Seydoux, Melissa Teixeira Magalhaes, Charles Bonsack, Philippe Golay, Danièle Spagnoli, Valentino Pomini

**Affiliations:** a Community Psychiatry Service, Department of Psychiatry, Lausanne University Hospital and University of Lausanne, Lausanne, Switzerland; b Institute of Psychology, Faculty of Social and Political Science, University of Lausanne, Switzerland

**Keywords:** Supported employment, Personality disorders, Mental disorders, Vocational rehabilitation

## Abstract

**Aim:**

People with severe mental illnesses (SMI) face different occupational challenges than those diagnosed with personality disorders (PD). Supported employment (SE) has been validated for SMI patients but its effectiveness for individuals with PD remains unclear, and the reasons for this potential difference have not been explored. This study aimed to identify differences in SE practice for clients with SMI and those with PD.

**Methods:**

Six SE job coaches were interviewed about their experiences. A thematic analysis was run.

**Results:**

More difficulties and facilitators were mentioned regarding clients with PD than regarding clients with other SMI. For both, patients’ symptoms were reported to negatively affect their (re)integration into the job market. However, in contrast to that of clients with SMI, the relation between symptoms and SE success for clients with PD involved difficult behaviors and their negative impact on the therapeutic relationship.

**Conclusion:**

In summary, SE practice seems to be undermined by PD and could benefit from adaptations, such as specific training for SE teams to help them in managing clients with this disorder.

## Highlights

• Patients' symptoms negatively affect their (re)integration into the job market.
• Job coaches have more difficulties with people diagnosed with personality disorders.
• Challenging behaviors of people with personality disorders impact the intervention.

## 1 Introduction

Mental disorders hamper employment. In Switzerland, half of all recipients of disability benefits qualify due to mental illness [1]. Of patients with schizophrenia and personality disorders (PD) in Switzerland, 80 % are unemployed and 50 % of those who are employed face problems at work [2].

Work issues vary according to the nature of the mental disorder. On the one hand, people with severe mental illness (SMI; e.g., schizophrenia, severe mood disorder), defined by their chronic characteristic of psychotic or mood symptoms, and high rates of relapse, affecting social and professional functioning, experience difficulties related to their health issues which, notably, results in a high rate of absenteeism [3]. Working full-time could worsen their symptoms [4]. Public and self-stigma toward their conditions hamper access to work [5,6]. Finally, they show cognitive impairments, notably regarding executive functioning, which induces poor performance and work quality [7,8].

On the other hand, according to the ICD-11, PD are characterized by problems in functioning of aspects of the self, and interpersonal dysfunction, manifest across a range of personal and social situations in patterns of cognition, emotional experience, emotional expression, and behavior, that are maladaptive [9]. DSM5's definition is similar, attesting of one or more pathological personality traits, and moderate or greater impairments in personality functioning, that are relatively inflexible and pervasive across a broad range of personal and social situations [10]. PD used to be classified categorically, but in recent decades, its definition has been moving toward a dimensional understanding, based on the fact that personality traits are constitutive of each person in a more or less problematic degree, reaching pathological level beyond a certain threshold [[11], [12], [13], [14]]. PD represents about 12 % of the general population [15] and 25–92 % of the psychiatric population [13,16,17]. Comorbidities are very common for this disorder whether they be disorders within the same category—personality disorder—in 60 % of cases [18] or other categories of disorders—including those recognized as SMI: About ¾ of people diagnosed with borderline PD (BPD) have a comorbid disorder other than PD [19]. Nevertheless, people diagnosed with PD have in common that they may struggle more with interpersonal difficulties, impulsivity, work conflicts (often resulting in intentional job loss), dismissals, demotion, and unemployment [[20], [21], [22], [23]]. PD's typical characteristics of relationship issues, difficulties in admitting one's own mistakes, mood swings, and resistance to instruction are seen as the person's fault in a work setting, unlike those of other psychological disorders that are considered to be illnesses and which trigger compassion. Thus, employers are particularly critical of staff members presenting PD characteristics [24].

Work increases the well-being of people with mental disorders [25]. Thereby, supported employment has been developed to help psychiatric patients to regain and maintain competitive employment. In this time-unlimited intervention, job coaches support patients at every step of vocational rehabilitation, according to their needs and preferences. Individual Placement and Support (IPS) is currently the most validated model of supported employment (SE) [26], particularly for people with SMI, for whom it was originally designed [27]. Only a few studies have explored this model's effectiveness for people with PD, with mitigated results. Juurlink, Lamers [28], Juurlink, Lamers [29] were the first researching this matter and found no significant difference in the results between IPS traditional clients and those with PD. However, this study had a small sample size, and the heterogeneity of the PD group could explain the intergroup equality. Whereas Dunand, Golay [30] compared PD groups according to different clusters and found that clusters A and especially B had poorer outcomes, notably in terms of rate of professional (re)integration into the job market, as well as time to reach employment, as compared to people in cluster C PD or without a PD. More research is needed to come to precise conclusions [[28], [29], [30]]; In that sense, Chanen, Nicol [31] are currently leading a randomized controlled trial on the effectiveness of IPS for young people with BPD. Most importantly, the reason for this potential difference in effectiveness remains largely unexplored. The clinical statements of SE coaches attest to difficulty following up with clients with PD of all types. Still, there is a lack of literature on this topic, except in other settings, where negative attitudes of health care staff toward people with PD [32,33] and psychosocial impairments of this population [34] have been shown, which could explain these individuals' lower rates of success in rehabilitation. As people with SMI do not present the same work-related impairments that people with PD do, and that professionals report more difficulties dealing with PD patients, this study aims to explore the nature of and contrasts between difficulties SE coaches face with these two populations, and what solutions to these issues can be considered.

## 2 Materials and methods

### 2.1 Procedure

We conducted a qualitative study with SE job coaches at RESSORT, a community network program for supported employment embedded with the Community Psychiatry Department of Lausanne University Hospital (Switzerland). SE was implanted at RESSORT in 2009, has followed close to 700 patients since then and has a cohort of around 60 patients at any given time. A part of its members has then been trained to the model by a team supervised by the founders of IPS in Montreal (Canada). RESSORT's coordinator was herself trained as an IPS supervisor. Since then, the initial team members internally train co-workers with course material validated by the IPS founders. The specificity of RESSORT SE team is that it is part of the hospital's public services. The treatment team is not directly attached to the service. Instead, anyone diagnosed with a mental illness and being treated by a psychotherapist is allowed to join, and job coaches are in regular contact with them. Compared to IPS standards, it implies that patients from all walks of life can participate, and that the level of required integration of treatment services depends on the goodwill of external psychotherapists. In addition, the model is influenced by Switzerland's economic context, in which the labor market is much less liberal than in the USA, where IPS was created. As a result, this team has a fair fidelity to the original model according to the IPS fidelity scale [35]. The Research Ethics Committee of the University of Lausanne approved the protocol (number #E_SSP_102020_00008).

### 2.2 Participants

Through our existing collaboration with the service, we invited SE job coaches from RESSORT in Lausanne (Switzerland) to participate. We aimed to reach data saturation, relating to the point where data repeats itself through interviews [36], and usually emerging between six and twelve participants [37]. Experience-related research questions require small to moderate sample size, in order to maintain the focus on individual experience, whilst obtaining patterns across the data set [38]. It is the case in the present study which enabled participants to share their personal experiences and reflect on concrete examples encountered in their practice, to enrich our understanding of this new topic. We therefore initially selected six coaches—out of the eight in the team—who agreed to participate and signed informed consent forms, and we reviewed their patient cohorts to ensure that they had had recent contact with individuals with both categories of disorders of interest to our study, with the possibility of adding more participants if saturation was not reached. When interviews were conducted, researchers sensed informational redundancy across participants and no more new themes and sub-themes emerged as of the fifth interview. We therefore decided to stop sampling.

In total, we interviewed six White job coaches, including two men and four women, between December 2020 and January 2021. Two of them were nurses, two occupational therapists, one a social worker, and one a psychologist. The average age was 38 years old (range: 31–50), number of years of experience as a job coach was 3 (range: 1–6) and number of years of experience in psychiatry prior to their current job was 8 (range: 0–23). Their average work rate as SE job coaches was 50 % (range: 20–80), with around 15 to 20 patients for a full-time position, of which approximately one third have a PD.

### 2.3 Measures

The second and third authors conducted semi-structured interviews based on a topic guide that included questions about coaches' experience with people diagnosed with SMI and PD. As this study was exploratory, we decided not to distinguish between different forms of SMI or PD, in order to capture a general idea of the difficulties encountered by SE job coaches. Moreover, the latter are not trained in specific mental disorders and are not necessarily familiar with the patient's precise diagnosis. Besides, studying PD as a whole is consistent with the recent dimensional model of PD, which involves examining a broad factor, referred to as the level of personality functioning, in terms of both self and interpersonal aspects [10]. In this view, different PD share common symptoms. For each category of patients successively, coaches were asked (a) what kind of difficulties they encountered, (b) which solutions they implemented or conceived for these issues, (c) if SE seemed adapted for the population, and (d) if they could consider potential adaptations to the model. To avoid possible order-effect bias or fatigue bias as the interview progresses, known in the field of surveys [39,40], three participants were assigned to a topic guide addressing SMI first and the other three were assigned to one addressing PD first. For this paper, we mainly focused on the difficulties and solutions mentioned and briefly discussed the adaptability of SE for different types of disorders.

Interviews took place in the participants’ office building and lasted around 1 h. Interviews were audio-recorded, transcribed, and anonymized. Pseudonyms were assigned to each participant.

### 2.4 Data analyses

The interviews’ content was thematically analyzed [38]. The first author coded the interviews into meaningful chunks, which were then collated and gathered into codes, from which themes and sub-themes were generated to organize and make sense of the data. Those were reviewed with the second and third authors until consensus, to increase interpretation objectivity. Additionally, the frequencies of each sub-theme and code were reported to describe their representativeness for the sample [41]. Sub-themes and codes were considered “general” when mentioned by all participants, “typical” when mentioned by five or four participants, “variant” when mentioned by three or two participants, and “rare” if mentioned by one participant.

## 3 Results

Two main questions were answered throughout the dataset: (1) the nature of difficulties SE coaches face with SMI versus PD patients, which refers to patients' characteristics, symptoms or behaviors, and problems in the therapeutic relationship or with the efficiency of the intervention as the negative outcomes such factors cause, and (2) facilitators SE coaches report with SMI versus PD patients*,* defined as strategies used by the coaches to overcome these difficulties, ideas of ways to improve the intervention, and present characteristics in patients that ease the intervention. They are not always available with every client but are features that greatly help coaches when present. However, in this study, most facilitators were related to the coaches’ practice rather than skills of the patients themselves.

One main finding is that the number of difficulties and facilitators mentioned by the job coaches—as counted as the codes—was considerably higher for people with PD than for those with other SMI. Moreover, many were general and applied to both categories of disorders. In total, 41 difficulties in SE intervention were identified, of which 25 applied mostly to people with PD, six mostly to people with other SMI, and 10 equally mentioned for both groups. Thirty-one facilitators were identified: 14 mostly for people with PD, three applicable only to people with other SMI and 14 equally shared by both groups. The difficulties were also qualitatively different between the two groups of disorders, which is reflected within the themes and sub-themes.

Question 1 Nature of difficulties SE coaches face with SMI versus PD patients

**Fig. 1:**
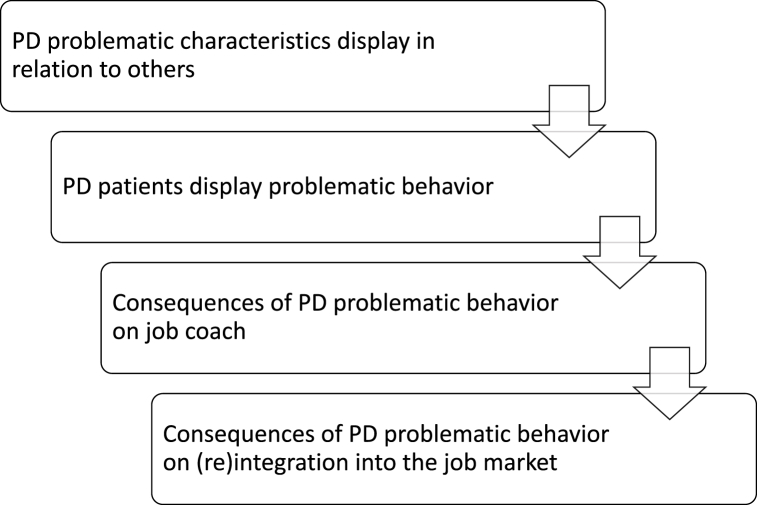
The Nature of and Links Between Difficulties Encountered by SE Job Coaches When Accompanying Clients Diagnosed With PD. *Note*. SE = supported employment; PD = personality disorder.

Theme 1.1 **Difficulties encountered with people diagnosed with PD in SE intervention are played out in relation to others.** The nature of difficulties linked with PD is described in Fig. 1. They were classified in four inter-related sub-themes. Table 1 details their composition.

**Table 1:** Sub-themes and codes of difficulties encountered with people diagnosed with PD in SE intervention are played out in relation to others (Theme 1.1).

Type of difficulty (sub-theme)	Difficulty described (code)
PD problematic characteristic display in relation to others (N = 6)	Emotional instability (N = 5; i.e. mood swings, impulsivity, disproportionate reactions)
	Lack of motivation (N = 5; i.e., lack of investment in the intervention)
	Ambivalence (N = 3; i.e., splitting, discrepancy between wishes and actions, instability of attitudes toward coaches and professional projects)
	Comorbidities (N = 3; i.e., other disorders diagnosed instead of PD, associated pathologies, disorders arising from PD)
	Rigidity (N = 3; i.e., resistance to change, unsuitability for the demands of the job market)
	Altered self-image (N = 2; i.e., lack of self-confidence, and accuracy of self-worth)
	Paranoia and sensitivity (N = 2; i.e., distrust, sense of persecution, vexation)
	Over-interpretation^a^ (N = 4; i.e., misinterpretations, exaggerations, taking things personally)
	External locus of control^a^ (N = 3; i.e., deflection and excuses-making)
PD patients display problematic behavior (N = 6)	Interpersonal difficulties (N = 6; i.e., lack of social barriers, revenge behavior, relationship testing, manipulation, perversion, imposition, idealization, devaluation, maladaptive attachment, lies, disrespect, contempt, dependance, opportunism)
	Conflicts (N = 5; i.e., confrontation, pushing to the limits)
	Project multiplication (N = 3; i.e., dispersal, difficulties following up on one project until the end)
	Avoidance (N = 2; i.e., escaping when change is about to happen, disappearance)
	Challenged framework (N = 2; i.e., intervention setting testing, rules negotiations)
	Project sabotage (N = 2; i.e., refusal to accept help, backtracking when change is about to happen)
	Triangulation (N = 2; i.e., splitting between job coaches and caregivers)
	Lack of commitment^a^ (N = 3; i.e., lack of proactivity in the intervention, idealized expectations toward job coaches, difficulty honoring commitments)
	Missed appointments^a^ (N = 2; i.e., regular (unjustified) delays and absenteeism)
Consequence of PD problematic behavior on job coach (N = 6)	Fatigue (N = 6; i.e., energy-consuming, emotionally involving, feeling of carrying everything on their own, constant work, frustrations)
	Particularly challenging intervention (N = 6; i.e., complicated follow-ups)
	Hypervigilance of the client's expression (N = 5; i.e. particular attention not to upset clients)
	Mixed feelings about the client (N = 3; i.e., interesting work at the same time as negative anticipation before the session because of clients' unpleasant attitudes)
Consequence of PD problematic behavior on (re)integration into the job market (N = 2)	Unemployment and complexity in maintaining professional activity (N = 2; i.e., difficulty to keep jobs)
	Futilely long SE intervention (N = 2; i.e., long follow-ups without any outcome)
	End of SE without being employed^a^ (N = 2; i.e., majority of failures to find employment)

*Note*. PD = personality disorder; SE = supported employment.

a Sometimes present in SMI but mostly in PD.

Most difficulties—like emotional lability, ambivalence, rigidity, and their way of perceiving self, others, and events—are associated with the patients themselves and are characteristics of PD. Patients with PD tend to interpret events in a way that quickly triggers them and affects their mood, which influences the optimal course of the intervention. A participant described all these aspects:

Normally, the client is also supposed to do things between two appointments, I don't know, for example, a client calls the companies to find out why they haven't been hired. And it's things that weren't being done, when he'd said yes, then he'd say “it's no use, because anyway …”. The difficulties were very much projected onto the outside world. “They're all idiots in this company”, “there's no point”. (Monica)

This results in some problematic behaviors that are also typical of PD, such as interpersonal difficulties, conflicts, challenging boundaries, avoidance, sabotage, and triangulation. A participant gave an example of these behaviors that bring problems into the intervention itself and the job search:

I had another patient who jumped from one project to another. It was hard to stay focused on one. We’d start and all of a sudden, he’d move on to something else because he had an idea in mind, because all of a sudden, he’d say “ah, this isn’t working, so I’m moving on to something else”. So it was a bit difficult to keep him focused on what we’d said to each other. (Daniel)

Clients’ problematic behaviors negatively impact their coaches, who must carefully choose their words when interacting with them. They often feel tired of taking care of them, which they found particularly difficult and unpleasant. This arduous work was illustrated by this participant:

What is complicated is to always be very attentive to the words chosen, it’s quite demanding to always weigh well the use of each word and to feel in what emotional state they are, to see how far we can go in what we say, in what we do, in the demands we make, in the way we give feedback on their behavior, on what they might have said during an interview or a phone call, or the way to write a letter. (Emilie)

This, in turn, hinders the efficacy of SE intervention, with long follow-up periods that do not necessarily end in employment. This participant showed how an intervention can last without being productive when patients stay stuck in one orientation that does not seem to work for them:

Even if we set goals every 3 months, if it’s been more than 10 months and nothing has changed and we’ve always set the same goals because the person didn’t want to open up employment opportunities, well, at some point, even if the IPS model says it’s good to continue, well, it becomes more difficult for the coach. (Max)

**Fig. 2:**
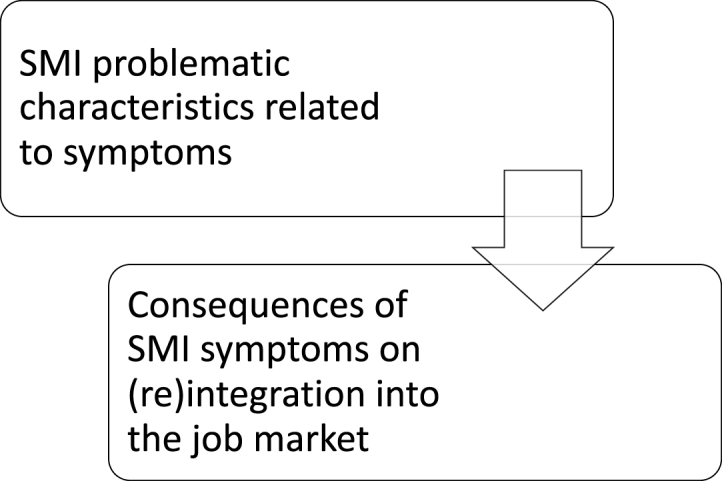
The Nature of and Links Between Difficulties Encountered by SE Job Coaches When Accompanying Clients Diagnosed With SMI. *Note*. SE = supported employment; SMI = severe mental illness.

Theme 1.2 **Difficulties encountered with people diagnosed with SMI in SE intervention related to their level of illness.** The nature of difficulties linked with SMI is described in Fig. 2. Its components are visible in Table 2.

**Table 2:** Sub-themes and codes of difficulties encountered with people diagnosed with SMI in SE intervention related to Their level of illness (Theme 1.2).

Type of difficulty (sub-theme)	Difficulty described (code)
SMI problematic characteristic related to symptoms (N = 6)	Functional limitations (N = 4; i.e., disorganization, attention problems, memory disorders, mistrust, difficulty entering into a relationship and communicating, lower work capacity)
	Anosognosia (N = 2; i.e., time needed to realize and accept weaknesses)
	Unstable health^a^ (N = 4; i.e., symptom recrudescence, work is not always the priority)
	Symptom visibility^a^ (N = 2; i.e., looking odd to others)
Consequence of SMI symptoms on (re)integration into the job market (N = 4)	Discouragement in case of failure (N = 3; i.e., difficulty staying motivated over the long term in the absence of success)
	Complexity in the work application process (N = 2; i.e., lack of autonomy, organization and rigor during job search)

*Note*. SMI = severe mental illness; SE = supported employment.

a Sometimes present in PD but mostly in SMI.

In comparison with PD, no difficulty described for people with other SMI was general to all coaches. No symptom characterizing SMI resulted in problematic behaviors. Rather, these patients are affected by health issues and symptoms, for example, problems in communication, memory, consistency, or organization. This participant showed such impairment's challenge for finding employment:

[A client] is particularly very, very, very disorganized, and therefore the slightest task takes him half a day. So we had to work on this capacity of disorganization first, which resulted in the fact that within a year and a half of follow-up, we were able to send only three job applications, because within the time he managed to prepare his application, well, the job advertisement did not exist anymore. (Emilie)

The therapeutic relationship is apparently not affected, unlike with people with PD. However, these difficulties have consequences on reaching employment and on both the coach and the patient, who become slowly discouraged. One participant explained this discouragement from both sides when no employment materializes.

When someone with depression is told “no” 20 times, after a while he says, “I don’t feel like it anymore.” Even you say to yourself, “I don’t feel like it anymore,” but for a person with depression, it’s even worse. (Daniel)

Theme 1.3 **General difficulties encountered in SE intervention for both PD and other SMI clients.** General difficulties are less in relation to patients' symptoms than to external constraints. This is shown in Table 3.

**Table 3:** Sub-themes and codes of general difficulties encountered in SE intervention for both PD and other SMI clients (Theme 1.3).

Type of difficulty (sub-theme)	Difficulty described (code)
Professional difficulty (N = 5)	Job market (N = 4; i.e., competition, job field influences success, influence of candidate's age and career path, COVID-19 resulted in home-office which is not adapted to certain patients)
	Career path (N = 3; i.e., dismissals, gaps in the resume, professional instability, inconsistencies in the career path, poor work certificates)
	Challenge of practicing job coaching with a psychiatric population (N = 3; i.e., finding a job necessarily more difficult when psychiatric symptoms are present)
Health difficulty (N = 5)	Crisis situation (N = 3; i.e., suicidal ideation, conflicts with peers, risk being expelled from the country)
	Negative impact of failure in reaching employment (N = 3; i.e, impact on hope, motivation, self-esteem, and self-confidence)
	Disability (N = 2; i.e., lower work capacity)
Coach's difficulty (N = 5)	Ending the intervention (N = 5; i.e., difficulty assessing when the intervention still makes sense for the client)
	Admitting that no miracle solution exists for dealing with complex cases (N = 2; i.e., even the best strategies and interventions are not always beneficial, some difficulties are out of their hands)
Patient's problematic behavior (N = 3)	No career goal (N = 2; i.e., absence of goal hindering the possibility of concrete steps toward change)
	Commitment irregularity (N = 2; i.e., clients' motivation and availability fluctuate with life events)

*Note*. SE = supported employment.

For example, strict requirements of the work market that demand skills, experience, flexibility, or initiative are an obstacle to finding a job. These make the work market difficult to reach in general, and even more in psychiatry. One interviewee explained that SE participants often aim at jobs in the same field, which makes it difficult to find positions for everyone: “There are certain professions that we accompany a little more in SE, and these professions are the ones that are a little more of dead-end jobs” (Max).

Some codes are related to the patients’ situation, and they affect their professional success but are not described by the coaches as elements that incur difficulties for the coaches in their tasks. This is the case for patients who are going through objectively difficult social events, those who must deal with job rejections, or those who are recognized as disabled, as this participant explained:

Sometimes they can work at a certain rate [ …] but they have a reduced output. [ …] Let’s say a worker works full time in a factory, that’s an example, so they can work at 100%, only they are a little slower [ …] so instead of producing a hundred bottles a day, they are going to produce seventy. The bottles will be beautiful, but they won’t produce the hundred bottles they’re supposed to if they were 100% profitable. (Angela)

On their side, coaches sometimes feel cornered or out of resources when it comes to ending an intervention that is no longer necessarily justifiable with a client for whom no employment perspective seems possible at that stage. They are afraid of shattering clients’ hopes. However, these coaches must sometimes accept that no magical solution exists and that certain situations may persist independently of their efforts and goodwill. This participant illustrated the consequences of ending interventions with some clients, showing why it can be so difficult:

SE intervention was clearly not possible, but when I had to make the decision to put an end to this follow-up, the patient did not agree at all, because for him it was really the only thing that kept him going. And since I announced that we were stopping, well, he stopped his medication because for him it didn’t make sense to continue taking antipsychotics if there was no prospect of a job. So, he’s slowly decompensating because he’s no longer medicated. (Emilie)

Coaches described a lack of career objectives and of regular participation in the intervention as the only problematic behaviors that were difficult for their mission and which applied to patients independently of their diagnoses. Indeed, it is common in psychiatry that patients miss appointments and disengage from interventions. This does not allow for optimal work to arise and therefore deteriorates SE success. One participant showed their need to always stay proactive to ensure that their client did not disengage from the intervention:

Sometimes they don’t say: “yes, we’ll see each other next week,” they say: “yes, we can see each other in 3 or 4 weeks,” and it’s up to us to be a bit vigilant, I think, to keep the focus, and to keep the rhythm. (Monica)

Question 2 Facilitators SE coaches report with SMI versus PD patients

Theme 2.1 **Facilitators of coaches and clients diagnosed with PD in SE intervention.** Most facilitators mentioned in the interviews were related to PD clients. They are visible in Table 4.

**Table 4:** Sub-themes and codes of facilitators of coaches and clients diagnosed with PD in SE intervention (Theme 2.1).

Type of facilitator (sub-theme)	Facilitator described (code)
Coach's facilitator with PD (N = 6)	Scientific research (N = 4; i.e., search of evidence-based strategies and tools)
	Collaboration with experts (N = 3; i.e., collaboration with PD experts for supervision, diagnosis assessments and who could refer them their patients who are ready to work)
	Communication style (N = 3; i.e., being clear and not afraid of naming things)
	Team discussions (N = 3; i.e., team reflections to find strategies)
	Inclusion of client's family (N = 2; i.e., family as extra support)
	Spontaneous strategies (N = 2; i.e., adapted natural attitude rather than planned strategies)
	Therapeutic distance^a^ (N = 6; i.e., avoiding feeling personally overwhelmed by situations)
	Regular questioning of the added value of SE for each patient^a^ (N = 4; i.e., set regular goals and their evaluation with the care network to avoid becoming stuck in an intervention that no longer makes sense for the patient)
	Professional experience^a^ (N = 3; i.e., experience in psychiatry, supervisions)
	Client's job support^a^ (N = 3; i.e., importance not to think that support is not needed once on the job)
	Intervention-time limitation^a^ (N = 2; i.e., research has shown that few jobs are found by persisting in SE beyond 9 months)
	Transparency with client^a^ (N = 2; i.e., show honesty and own doubts)
PD skill (N = 3)	Ease in starting the intervention and looking for a job (N = 3; i.e., relative ease in the process of application)
	High cognitive skill level (N = 2; i.e., fair level of education, comprehension and intelligence)

*Note*. SE = supported employment; PD = personality disorder.

a Sometimes present for SMI but mostly for PD.

Almost all the facilitators that exist or are possible in interventions with PD clients are strategies set by the coaches. Such strategies rely on various forms of support: from science, experts in the disorder, coaches' teams, and clients' families. PD obviously reinforces the coaches' need for professional community. Coaches also decide to adopt a certain approach toward clients with PD, expressed in the coaches' manner of communicating, distancing themselves, or setting boundaries around the support they can bring. This was reflected in one participant's discourse:

I reread one of his cover letters and made proposals. In fact, every time I made a proposal, he would justify for three minutes why he had done it that way, etc. In other words, I couldn't offer him anything because his letter was perfect and he shouldn't have touched it. [ …] So I said to him: "Well then, fine, we'll leave it like that if you think it's perfect. There's no problem. But my role is to check and try to improve it. For me, by putting these things in, it's improved. If I put myself in the place of an employer, I'd like it to be more like that than like you've done it, which doesn't mean that what you've done is bad. Because as many people are going to read your letter, as many people are going to give you their opinion, I say, but at the same time if I don't give you my opinion, I'm useless". So then he said “no, no, no, but it's okay, do it”. So he let me do it, but it took a bit of readjustment. So sometimes, you get into things that are a bit subtle like that, and you have to take it on the fly and then try a strategy that doesn't always work, but that worked, so much the better. (Astrid)

Only two facilitators out of the 13 coaches mentioned in relation to their clients with PD were linked to those clients' skills. Furthermore, these two facilitators were considered “variant”. As opposed to people with SMI, people with PD were described as being able to sell themselves well to find a job and having high levels of intelligence and education; as one participant noted, their clients with PD “are often people who have managed to study quite far, so that’s not going to prevent them from finding a job” (Max).

Theme 2.2 **Efforts furnished by clients diagnosed with SMI as a facilitator of SE intervention.** Only a few categories of resources were linked to SMI patients. They are presented in Table 5.

**Table 5:** Sub-themes of efforts furnished by clients diagnosed with SMI as a facilitator of SE intervention (Theme 2.2).

Facilitator described (code)
Adequate understanding of one's disorder of people with SMI (N = 3; i.e., symptoms recognition and management)
Ease of maintaining a job for clients with SMI (N = 2; i.e., reliable and pleasant workers)
Confronting clients with SMI (N = 2; i.e., let patients become aware of their limitations through their experience and by showing them transparency)

*Note*. SE = supported employment; SMI = severe mental illness.

Most facilitators described in SE intervention for people with SMI were related to those clients' own skills, such as their capacity to understand, anticipate, and control their symptoms or—unlike people with PD—to keep a job, even though finding one is a challenge. A participant explained how this knowledge about one's disorder is an argument that helps present a client to a company as a potential hire: “It's a tool that can also be used with employers, to say: ‘he knows his limitations, thus he can anticipate [his reactions]’” (Angela).

Only one variant code related to coaches’ strategies. It consists in managing patients experiencing anosognosia by allowing them to reach a certain work rate or work domain with which their limitations are considered incompatible. Thereby, such clients become aware of their weaknesses, which helps them move away from unattainable careers to more suitable ones.

Theme 2.3 **General facilitators in SE intervention for both PD and other SMI clients.** General facilitators refer to strategies that coaches (would like to) use to improve the intervention, or to components that are external to both parties involved in the therapeutic relationship, and yet which ease the job coaching. They are shown in Table 6.

**Table 6:** Sub-themes and codes of general facilitators in SE intervention for both PD and other SMI clients (Theme 2.3).

Type of facilitator (sub-theme)	Facilitator described (code)
Coach's general facilitator (N = 6)	Setting boundaries (N = 6; i.e., keep focus on goals)
	Teamwork (N = 6; i.e., well-functioning care network, support of the team and supervisions as resources)
	Referring patients outside of SE (N = 5; i.e., in case of failure with the model)
	Inter-individual differences between job coaches (N = 4; i.e., different sensitivity, ease and opinion in the team as a strength)
	Shared responsibilities (N = 4; i.e., mutual agreements, empowering clients without burdening them)
	External sources of support (N = 3; i.e., relaxing activities outside of work, personal introspection)
	Continuous training (N = 3; i.e., training about different disorders, suicidal ideation management and more)
	Meta-communication (N = 3; i.e., directly rephrasing what is happening, what has been said with patients, and communicating with colleagues about how they feel)
	Humor (N = 2; i.e., use of humor with the team and the patients, when the alliance allows, to dedramatize situations)
	Therapeutic alliance (N = 2; i.e., enables a beneficial intervention through trust and an adapted mode of communication once established)
Professional facilitator (N = 4)	Professional network (N = 4; i.e., SE systematic job development, word of mouth with colleagues and patients' string-pulling)
	Employers' sensitivity (N = 2; i.e., raising awareness about mental illness among employers)
	Rethinking the job market (N = 2; i.e., moving toward a social economy, seeking more help from the government)
	Strategies in choosing targeted job (N = 2: i.e., trying to avoid stressful or dead-end domains)

*Note*. SE = supported employment.

The first sub-theme of general facilitators was composed of codes like that of coaches’ facilitators related to clients with PD. Indeed, all participants valued teamwork—

including their colleagues and the added value of each—, supervision, training, collaboration with all other actors involved in the clients' recovery or transferring clients to other institutions when needed. Appropriate opportunities for collaboration were not always present, but the best outcomes were noticed by the coaches to correspond with their “hand-in-hand” work with other professionals. Additionally, coaches chose to implement strategies which entailed specific behaviors, such as setting clear boundaries, sharing responsibility with the client for the intervention's goal, meta-communicating to avoid misunderstandings or misinterpretations, or using humor both with clients and to put difficulties into perspective with other professionals. One participant explained how they involve clients in decision-making during the intervention:

I’m going to tell him that we’ve tried everything, and then what do we do with that, what does he suggest? In a way, give him back his responsibility, and not endorse all that myself—without burdening him either, because he shouldn’t feel guilty that we’re stuck. (Astrid)

Professional facilitators included the professional network of both the client and the coaches and components of the job market, such as employers' knowledge of and openness to mental disorders, and the professional sectors that were hiring or not. This participant described the lack of awareness about mental health in certain work settings, which makes it difficult to reintegrate patients:

We went to introduce the SE program to the human resources [department] of a big company. And my colleague added, “well, SE is also a program for severe psychological impairments,” but I think the HR manager immediately said, “but we don’t have any employees with severe psychological impairment.” I said, “maybe there’s someone who works for you at 50% and it’s not a choice, it’s a question of health, and they work very well at 50%.” (Angela)

## 4 Discussion

Results can be addressed from two different angles. Quantitatively, job coaches reported more difficulties working with clients diagnosed with PD than they did for those with other SMI. Further, those difficult experiences with clients with PD were more often shared across the group of job coaches in comparison with those reported for people with other SMI. Paradoxically, more facilitators were also mentioned regarding follow-ups with PD clients than with other SMI clients. This could be due to our facilitator concept not only including features that are actually present but also what helps when present and suggestions for improvement. It is also possible that fewer facilitators are necessary for coaches to successfully work with clients with SMI, while coaches expressed the need to be creative and use support with PD clients. Many of the mentioned difficulties and facilitators were generalized and applicable to all patients, regardless of diagnoses. This could be because the coaches' tasks are similar across their clients and the difficulties they might face and several components they must work with, such as the constraints of the job market, are the same independently of their clients' pathologies. Several of these difficulties were shared by most coaches. Moreover, coaches’ attitudes toward their clients and their approaches to practice, which underlie many of the facilitators mentioned, are likely to be their professional ethos, and therefore are similar with all clients.

Qualitatively, coaches reported the same difficulties that are mentioned in the literature. Issues regarding PD clients are more relational [[20], [21], [22], [23]], which seems to challenge coaches and impact interventions to a very significant extent. Difficulties mentioned for people with SMI were more often challenges for the clients themselves, such as managing their own symptoms [[3], [4], [5], [6], [7], [8]]. This obviously affects the effectiveness of the job coaching but does not seem to particularly challenge coaches and the therapeutic relationship.

Several PD characteristics seem to be externalized as problematic behaviors affecting others [42]. For example, emotional instability results in impulsivity, which can translate into conflicts, whether these are at work or with the clients' caregivers. A triggering event in a certain context will change the mood of a client with PD and affect other people in other contexts as well. Problems become shared between surrounding people instead of staying at a personal level. In short, PD problems are often large-scale and affect the client's whole environment instead of being internalized and contained, in comparison to those of people diagnosed with other SMI, who are more inclined to experience symptoms defined as internalized [43]. This might be explained by the fact that people diagnosed with PD have difficulties with self–other distinction [44]. Difficulties of people with SMI—such as discouragement—also affect coaches, but the internal conflict is not transferred into the therapeutic relationship. The personal nature of these patients' difficulties makes it easier for coaches to solve them. In comparison, if problems affect others, it adds layers of challenges for coaches, who must manage their own reactions and emotions, those of the other, and those at play in the therapeutic relationship.

In parallel with the identified difficulties, most facilitators mentioned for people with PD come from the coaches while those mentioned for people with other SMI are client-related. This does not mean that PD patients lack personal resources. In fact, their extreme traits are often exaggeratedly present qualities, which would benefit from being softened to serve as a strength and be valuable at work and even be highlighted when disclosing a condition to employers. This is the case, for example, with sensitivity, perfectionism, or vigilance, which become problematic only when they are ill-adapted in a context. It would be interesting to identify these traits at the beginning of the intervention and set their mitigation as an agreed-upon target for the job coaching [45]. Thereby, SE would still focus on impairments rather than on a diagnosis.

One of the only personal aspects mentioned as a facilitator for people with PD was their generally high cognitive skill level. This might generate expectations from the coaches, who see such clients as possessing qualities that are valued on the work market, making them forget about the emotional level of disability characterizing PD clients, and therefore reducing their tolerance for difficulties that appear during the intervention. This was not pointed out in the interviews. This raises the question of relevance of warning coaches about certain potential biases they could fall into.

This research enabled us to draw a contrast between the SMI and PD populations, and to gather coaches' opinions on the adaptability of SE to these groups, based on their experience. The suitability of the SE program was more often questioned by coaches for people with PD than for people with other SMI. Such uncertainty could seem logical, given the fact that SE was conceptualized for people with SMI and is not illness-oriented [46]. However, SE is flexible and should be suitable for, and adapting to any psychiatric population. This clinical statement might be due to coaches' lack of knowledge on how to deal with people with PD. Indeed, the SE project focuses on work impairments rather than on diagnoses. Coaches are not healthcare providers but social workers, who are not trained in the management of specific mental disorders, as patients' treatment is received outside of SE. While this helps combat stigma, coaches might not be adequately equipped to support the PD population. Furthermore, some SE requirements, such as adaptation to patients’ wills in terms of career goals and support time length, might even be detrimental for PD patients who need more structure [47].

This study completes and supports quantitative results regarding SE's lessened efficiency for people with PD, especially clusters A (i.e., odd, eccentric) and B (i.e., dramatic, erratic) who seem to reach poorer vocational outcomes regarding employment status, activity (i.e., job or education) maintenance, time before finding a job and type of income [30]. Facilitators that are described for people with PD are still compatible with the SE philosophy and most of them are already being used, which brings reassuring perspectives. Promising ideas of systematic change that could be implemented were mentioned, such as time-limiting the intervention when the support does not help the client—which corroborates the finding that if no job has been started after 9 months in SE, likelihood of finding employment drastically decreases [48]—regularly questioning the added value of the intervention, or developing collaborations with PD experts. These suggestions are in line with a set of guidelines of recommended therapeutic practices to accompany patients with BPD, called Good Psychiatric Management [GPM; 47]. This evidence-based approach condenses what works in specialized treatment for people with BPD and can be used by mental health professionals from any background. It consists in a general attitude rather than a structured intervention, and therefore can be relevant in different contexts of psychiatry. Like in SE, GPM is individualized, and work constitutes one of the main intervention goals. The GPM could meet the need for structure that SE lacks, as mentioned above. Several of its recommendations were already spontaneously used by the coaches, such as using their common sense, being transparent, sharing responsibility, or feeling and adapting to their clients' emotional states. One central point of this approach is to practice psychoeducation with patients, which is consistent with the idea of identifying extreme personality traits. In the sense of easing SE practice to better accompany people with BPD toward employment, it would be relevant to train coaches to the GPM approach, especially given the fact that they mentioned continuous training as something motivating and helpful.

To the best of our knowledge, this is the first qualitative study exploring SE coaches' perspectives on their interventions. However, the number of participants was limited and, even though we judged saturation to have been reached, this concept remains controversial in the qualitative research literature [36]. Again, as SE focuses on recovery, employment specialists do not necessarily have precise knowledge of their patients' mental illness and may, for example, have mentioned users with comorbid SMI and PD, thinking of the dominant disorder. This also explains why we chose broad categories of disorders to explore the difficulties described by job coaches, with the risk of inducing bias toward groups. Because of this choice, the interviews' topic guide directed coaches to consider people with PD or with SMI in general, without specifying a diagnosis, while we know that a wide range of disorders with their respective issues exist in each category, likely leading to a certain degree of approximation in the participants' responses. We cannot exclude the possibility that coaches answered our questions with some level of stigma they carry about mental disorders. Nevertheless, the interviewers asked participants for specific examples about the clients they had followed in their practice to limit this bias. Finally, this study was led in Lausanne, Switzerland, with its specific job market and situation, and where staff talk about challenges working with people with PD, although this attitude seems to be shared in other healthcare settings [32,33]. The authors adopted a reflexive posture throughout the study process, with the aim of reducing possible biases in the co-construction of meanings that such research involves. The first author is trained as a psychologist and works regularly with the job coaches. However, she is independent of the patients’ treatment. Analyses were also discussed with other members of the research team, including clinical psychologists, senior research psychologists and a senior psychiatrist, as well as at interdisciplinary meetings comprising clinicians, researchers, peer-practitioners and patients. The question of the extent to which the results can be generalized to all SE teams remains open.

## 5 Conclusion

SE practice seems to be undermined by PD. Therefore, we can confidently argue that adjustments in SE should primarily be made for PD, such as specific training for SE teams to care for people with the disorder as soon as they enter the program. For example, the implementation of the GPM for BPD [47] could be considered for implementation in the SE context. A recent implementation study shows promising results in this regard [49].

## Data availability statement

The authors do not have permission to share data.

## CRediT authorship contribution statement

**Noëllie Dunand:** Writing – review & editing, Writing – original draft, Resources, Project administration, Methodology, Formal analysis, Data curation, Conceptualization. **Marine Seydoux:** Writing – original draft, Project administration, Methodology, Formal analysis, Data curation, Conceptualization. **Melissa Teixeira Magalhaes:** Writing – original draft, Project administration, Methodology, Formal analysis, Data curation, Conceptualization. **Charles Bonsack:** Validation, Resources, Conceptualization. **Philippe Golay:** Writing – review & editing, Writing – original draft, Methodology, Conceptualization. **Danièle Spagnoli:** Validation, Resources, Project administration, Conceptualization. **Valentino Pomini:** Writing – original draft, Validation, Supervision, Resources, Methodology, Formal analysis, Conceptualization.

## Declaration of competing interest

The authors declare that they have no known competing financial interests or personal relationships that could have appeared to influence the work reported in this paper.
